# Isolated neutropenia as a rare but serious adverse event secondary to immune checkpoint inhibition

**DOI:** 10.1186/s40425-019-0648-3

**Published:** 2019-07-05

**Authors:** Abdul Rafeh Naqash, Ebenezer Appah, Li V. Yang, Mahvish Muzaffar, Mona A. Marie, Justin D. Mccallen, Shravanti Macherla, Darla Liles, Paul R. Walker

**Affiliations:** 1 Division of Hematology/Oncology, East Carolina University, 600 Moye Boulevard, Greenville, NC 27834 USA; 2 Brody School of Medicine, East Carolina University, 600 Moye Boulevard, Greenville, NC 27834 USA

**Keywords:** Immune checkpoint inhibitors, Neutropenia, Immune-related adverse event, C-reactive protein, IL-6, Immunosuppression

## Abstract

**Background:**

Compared to conventional chemotherapy, Immune checkpoint inhibitors (ICI) are known to have a distinct toxicity profile commonly identified as immune-related adverse events (irAEs). These irAEs that are believed to be related to immune dysregulations triggered by ICI can be serious and lead to treatment interruptions and in severe cases, precipitate permanent discontinuation. Isolated neutropenia secondary to ICI has been rarely documented in the literature and needs further description.

We report a case of pembrolizumab related severe isolated neutropenia in a patient with metastatic non-small cell lung cancer. We were also able to obtain serial blood and plasma-based biomarkers for this patient during treatment and during neutropenia to understand trends that may correlate with the irAE. In addition we summarize important findings from other studies reporting on ICI related neutropenia.

**Case presentation:**

A 74 years old Caucasian male treated with single-agent pembrolizumab for metastatic non-small cell lung cancer presented with fevers, chills, and an isolated neutrophil count (ANC) of 0 2 weeks after the fourth dose. In addition to antibiotics, due to the strong suspicion of this neutropenia being immune-mediated, he was started on 1 mg/kg of steroids and also received filgrastim to accelerate neutrophil recovery. Serial trends in C-reactive protein and certain other inflammatory cytokines demonstrated a corresponding rise at the time of neutropenia. Post recovery, his pembrolizumab was kept on hold. Eight weeks later he had a second episode of neutropenia which was again managed similar to the first episode. Despite permanent discontinuation of ICI after the first neutropenia, his disease showed an ongoing complete metabolic response on imaging. Our literature review reveals that hematological toxicities constitute < 1% irAEs with isolated neutropenia roughly accounting for one-fourth of the hematological irAEs. Based on the handful of ICI related neutropenia cases reported to date, we identified nivolumab to be the most common offender. The median number of ICI cycles administered before presenting with neutropenia was three, and the median time to recovery was approximately two weeks. All of these neutropenic episodes were ≥ grade 3 and led to permanent ICI discontinuation. Using immunosuppressive therapies in conjunction with granulocyte-colony stimulating factor was the most common strategy described to have favorable results.

**Conclusion:**

Neutropenia as an isolated irAE secondary to ICI is rare but represents a severe toxicity that needs early recognition and can often result in treatment discontinuations. Careful monitoring of these patients with the prompt initiation of immunosuppressive and supportive measures to promote rapid recovery as well as prevent and treat infectious complications should be part of the management algorithms. Serial monitoring of blood and plasma-based biomarkers from more extensive studies may help in identifying patients at risk for irAEs and thus guide patient selection for ICI.

## Introduction

Due to their ability to modulate certain inhibitory pathways, immune checkpoint inhibitors (ICIs) promote a T-cell mediated attack against tumor cells and thus harness the immune system to generate anti-tumor immunity. The recent advent of ICIs has radically altered the treatment approaches and revolutionized the outcomes for several tumor types that until recently were known to have dismal outcomes [1]. Non-Small Cell lung cancer (NSCLC) especially has witnessed a paradigm shift with significant improvements in survival, response rates, and durability of disease control, both in upfront and second line setting [2]. Based on results from Keynote-024 [3], single agent pembrolizumab was approved by the US Food and Drug Administration in 2018 in the frontline treatment of metastatic NSCLC having PD-L1 of ≥50%. Recently reported updated results from this trial show a 16-month overall survival benefit of pembrolizumab over platinum-based chemotherapy in patients with previously untreated, advanced NSCLC without *EGFR/ALK* aberrations. [4]

Compared to conventional chemotherapy, ICIs have been noted to display distinct patterns of immune toxicities, commonly labeled as immune-related adverse events (irAEs). IrAEs differ from usual toxicities in terms of having a more than likely immunological basis and can have a broad spectrum of manifestations that can involve different organ systems [5]. The incidence, distinct tissue specificity, timing, and severity of irAEs are variable and considered to be dependent on the type of ICI antibody and underlying malignancy [6]. In general, data from ICI related clinical trials and retrospective studies have indicated the incidence of irAEs such as colitis, pneumonitis, and thyroiditis to be higher compared to nephritis, myocarditis, or myositis. The incidence of the hematological adverse effects in general, and neutropenia, in particular, have rarely been documented as an adverse event secondary to ICI, with an overall reported incidence of < 1.0% [7]. A recent study that queried a World Health Organization (WHO) pharmacovigilance database (VigiBase) for ICI related hematological toxicities described autoimmune anemia and immune thrombocytopenia as the most common hematologic toxicities [8]. Conversely, a French pharmacovigilance study reported neutropenic irAEs to account for approximately one fourth (26%; *n* = 9/35) of all immune hematological irAEs [9]. In addition to these studies, to the best of our knowledge, there are nine other individual cases of neutropenia-related to ICI treatment published so far ([10–17]; Table 1).

**Table 1:** Summary of recently pubmished cases with neutropenia due to immune checkpoint inhibitors

Authors	Age, Gender	Cancer type	Immune checkpoint inhibitor	No. of cycles before neutropenia	CTCAE (4.03) Grade/ ANC Nadir /mm3	Rheumatological/autoimmune disease or serology	Duration of neutropenia	Bone marrow findings	Associated or preceding irAE	Treatment	Outcome	ICI restarted
Akhtari et al. [9]	42 Female	Melanoma	Ipilimumab	4	G4 ANC - 380	Antineutrophil and platelet autoantibodies.	60 days	Normocellular marrow, myeloid hypoplasia. Second bone marrow: hypocellular; granulocytic hypoplasia and lymphohistiocytic aggregates.	Rash	Pegfilgrastim Prednisone Dexamethasone IVIg, Cyclosporine	Prolonged neutropenia with multiple relapses. Recovery of counts after second course of IVIg. Developed anemia and thrombocytopenia	No
Ban-Hoefen et al. [10]	54 Male	Melanoma	Ipilimumab	4	G4- ANC - 0	No serology	52 days	Hypercellular; increase bland histiocytes, lymphocytosis; near complete absence of granulocyte precursors	Rash	Prednisone IVIG, Cyclosporine Filgrastim, Anti-thymocyte globulin (ATG)	Recovery of counts with ATG/cyclosporine/prednisone combination with filgrastim. Steroids tapered off after 4 months. Normal ANC after 6 months.	No
Tabchi et al. [11]	74 Female	NSCLC	Nivolumab	2	Severe neutropenia likely G-4 (ANC not reported)	Ulcerative colitis in remission	16 days	Absence of myeloid precursors	Hepatitis	Filgrastim, IVIG Prednisone Methylprednisolone	Responded to high dose methylprednisolone.	No
Wozniak et al. [12]	35 Male	Melanoma	Ipilimumab	3	G-4 ANC - 0	No serology	16 days	Granulocytes with features of rejuvenation and preserved maturation; poorly represented erythrocytes.	Rash	Methylprednisolone Filgrastim (both started after 8 days)	Recovery of counts 16 days.	N/A
Barbacki et al. [13]	73 Female	NSCLC	Pembrolizumab	2	G-4 ANC-0	Autoimmune myositis (in remission) Crohn’s disease	12 days	Not performed	None	GCSF, Methylprednisolone, IVIG, Cyclosporine	Recovered counts after 12 days. No recurrence at 3 months	No
Sun et al. [14]	64 Male	Prostate Cancer	Ipilumumab	2	G-3 ANC-770	Weak neutrophil reactive IgM antibodies	14 days	Not performed	None	Methyl prednisone followed by prednisone	Count recovery with no recurrent neutropenia. PSA remained undetectable and patient started lepurolide	No
Meti et al. [15]	59 Male	Melanoma	Ipilimumab/Nivolumab	2	G-4 ANC-0	No serology	16 days	Variable cellularity, hypoplastic granulocytic, unremarkable erythroid and megakaryocytic lineages.	Rash, hepatitis, colitis	Methylprednisolone, IVIG, filgrastim, mycophenolate mofetil (MMF).	Recovered counts after addition of MMF.	No
Turgeman et al. [16]	73 Male	NSCLC	Nivolumab	5	G-4 ANC - 0	Crohn’s disease No serology	7 days	Not performed	Diarrhea	Methylprednisolone, GCSF	Neutrophils started improving after a week..	N/A
	74 Male	NSCLC	Nivolumab	11	G4 ANC-0	Negative Serology	2 days	Mildly hypercellular, unremarkable erythropoiesis and megakaryopoiesis, hyperplasia of myelocytic precursors	None	G-CSF, prednisolone	Recovered ANC after 2 days. Developed multiple relapses. Placed on Erlotinib, prophylactic GCSF.	No

Abbreviations: *CTCAE* Common terminology criteria for adverse events, *ANC* Absolute neutrophil count, *G* Grade, *G-CSF* Granulocyte colony stimulating factor, *NSLC* Non-small cell lung cancer, *IVIG* Intravenous immune globulin, *ATG* Anti-thymocyte globulin

Results are presented as cumulative of all cases. The median number of ICI cycles before patients presented with neutropenia was 3. All most all patients had nuetropenia ≥ G3. Median time to resolution of neutropenia wa approximaely 2 weeks. Rash seemed to be the most common associated irAE that preceeded or occurred concurrently with neutropenia. Prednisone. IVIG and filgrastim were the most common modalites used in management with variable sequence of administration. None of the patients restarted ICI after resolution of neutropenia

We report a case of recurrent isolated severe neutropenia in a patient with metastatic adenocarcinoma of the lung treated with pembrolizumab. Coincidentally we were also able to obtain serial cytokine levels, and peripheral T-cell counts for this patient during his treatment and neutropenia as this patient was part of a study cohort with institutional approval for a biomarker study that allowed the collection of serial blood and plasma for relevant translational studies (ECU IRB 16–000719). Herein we also summarize essential findings from previously reported cases of neutropenia, discuss possible mechanisms contributing to this toxicity and elaborate briefly on management strategies that seem to work best for this toxicity.

## Case report

**Fig. 1:**
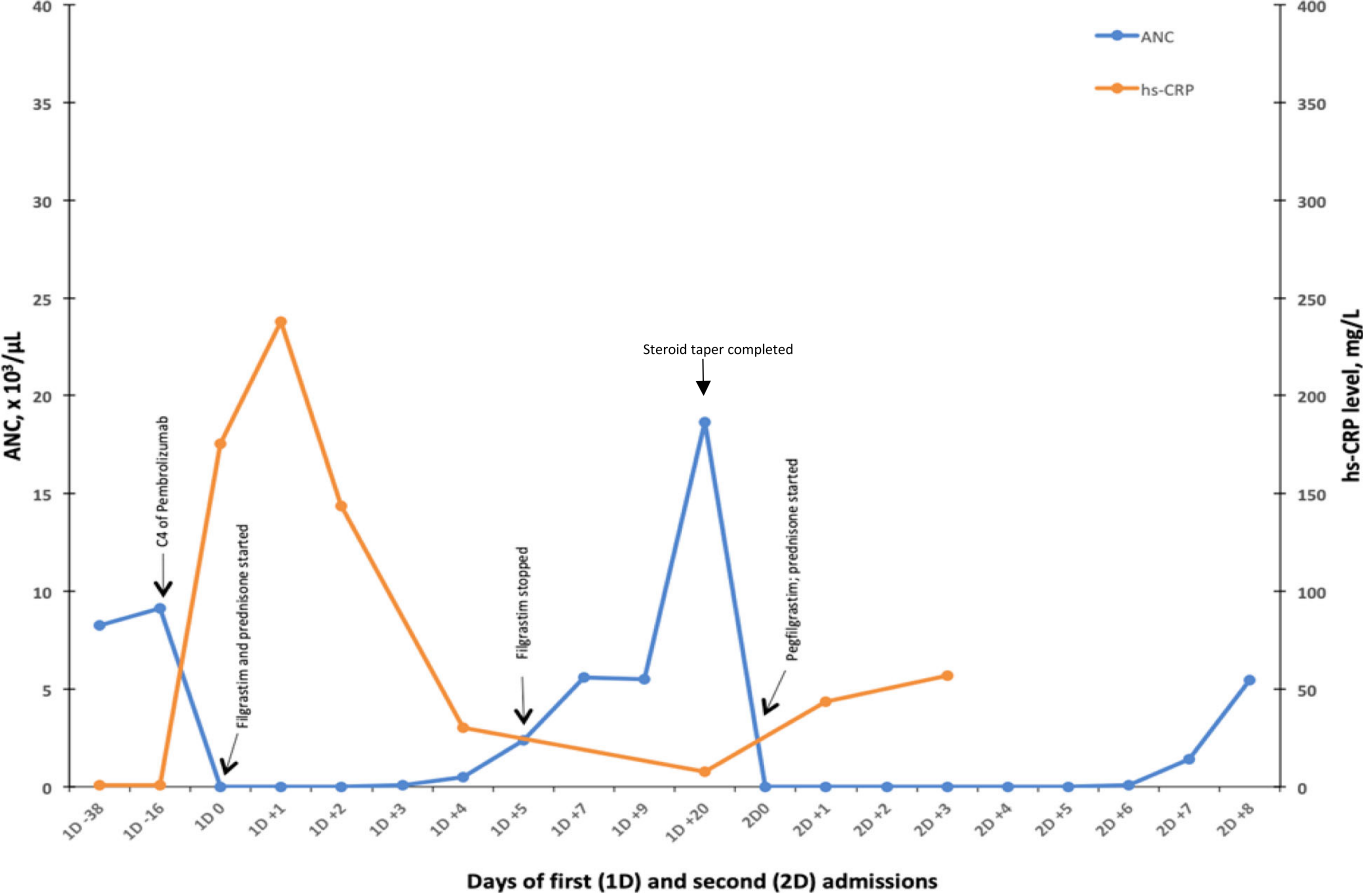
Graph showing the trend of the ANC and CRP for the first and second episode of neutropenia. 1D0 = Day 0 for first neutropenic episode, 2 D0 = Day 0 for second neutropenic episode. Corresponding days are measured based on days from the first neutropenic episode (1D0) and day of the second neutropenic episode (2D0). Note rise in CRP corresponds to fall in ANC in both instances. ANC improved with the use of filgrastim daily for 4 days at the first neutropenic episode and one dose of pegfilgrastim with the second neutropenic episode. Steroid taper for the first neutropenic episode was completed on day + 28 from neutropenia onset. ANC recovery in both episodes was observed 4 to 5 days from neutropenia onset

**Fig. 2:**
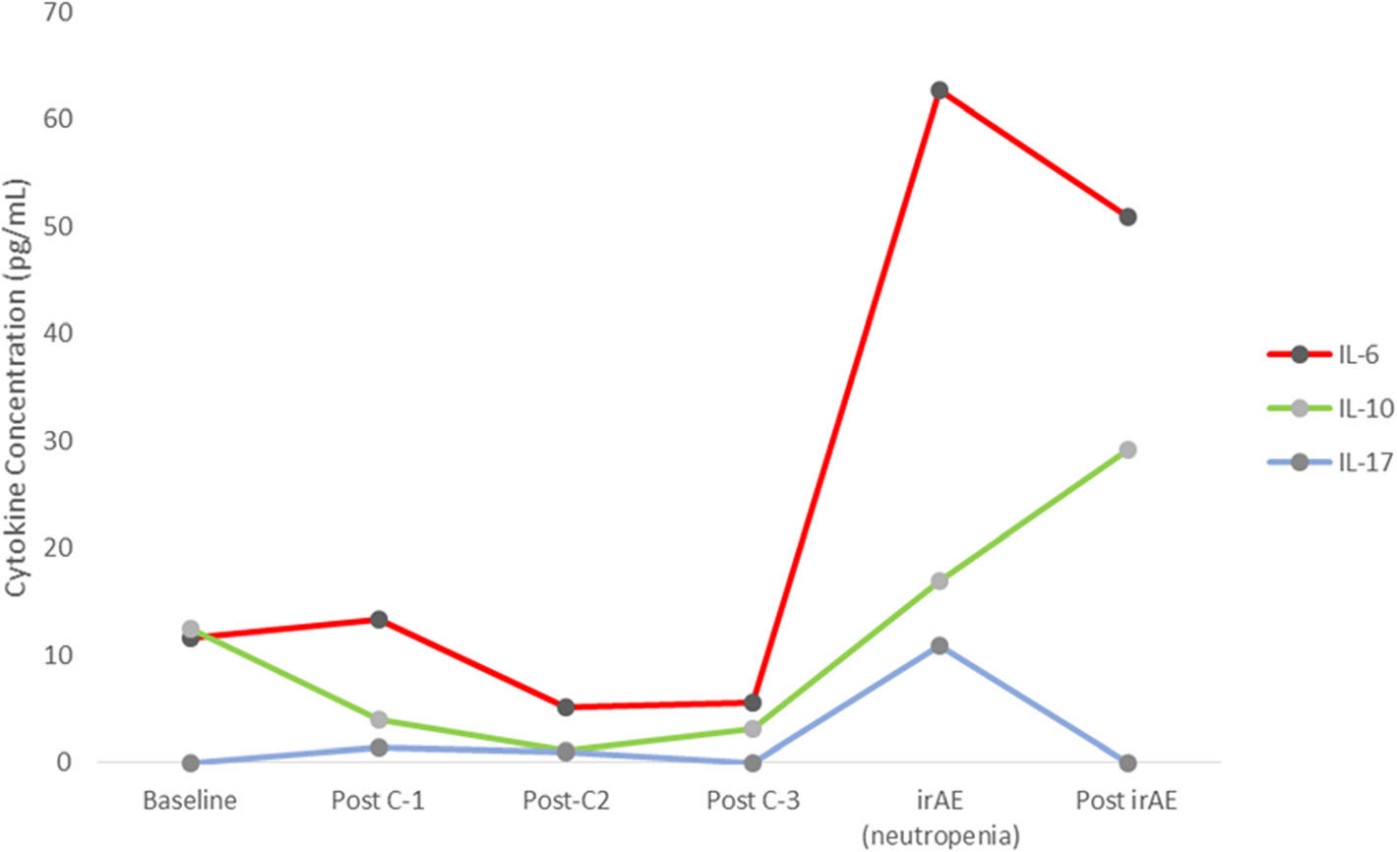
Cytokine concentration during the treatment course and at the time of neutropenia. Post-C4 levels are not displayed as the patient was admitted two weeks after C3, i.e., prior to C4 sample collection. Compared to baseline, a significant rise in IL-6, IL-10 and IL-17 are seen at the point of irAE. These co-relate with rise in CRP (Fig. 1). Two weeks post irAE, IL-6 and IL-17 levels demonstrate a downtrend while IL-10 level was noted to be rising. Sample collection at neutropenia was a day after treatment with steroids. Hence the treatment effect cannot be entirely excluded.

A 74-year-old Caucasian male with an Eastern Cooperative Group performance status of 1 and a 150 pack -year smoking history initially presented with progressive right upper extremity weakness. Further workup revealed a 1.4 cm frontal lobe mass on magnetic resonance imaging. Computerized tomography (CT) of the chest identified a spiculated mass lesion measuring 1.6 × 1.1 cm in the right hilar region. Apart from hypermetabolic activity in the lung mass, a staging positron emission tomography (PET) identified avidity in the mediastinal and hilar lymph nodes. Biopsy of the lung mass and hilar nodes identified moderately differentiated adenocarcinoma of lung origin. Based on this tumor size and nodal involvement, his intrathoracic disease was staged as IIIA (AJCC 7th). His solitary left precentral gyrus mass was treated with gamma knife radiosurgery, and he was subsequently placed on steroids with improvement in his limb weakness. His intrathoracic disease was treated with four cycles of cisplatin and pemetrexed with concurrent definitive radiation therapy. During follow-up, a surveillance PET scan approximately 11 months later was notable for new metastatic liver, mediastinal, para-aortic and right lower lobe lesions. Due to his original biopsied tissue having PD-L1 expression of 50% (22c3 antibody), he was started on single agent pembrolizumab 200 mg every 3 weeks. His baseline blood counts before starting pembrolizumab were all within the normal range. Two weeks after completing the fourth cycle of pembrolizumab, he presented to the emergency department with fever, chills, and general malaise. He was noted to be neutropenic with an absolute neutrophil count (ANC) of 0, which previously was noted to be normal the day of his fourth dose of pembrolizumab (Fig. 1). Hemoglobin was 12.6 g/dl, and platelet count was normal. The patient was hospitalized with febrile neutropenia and started on broad-spectrum antibiotics. As part of our programmatic approach, a serum C-reactive protein (CRP) level was obtained, which was markedly elevated at 175.4 mg/L (Fig. 1). He was started on prednisone 80 mg daily and filgrastim dose of 5 mcg/kg daily for 4 days. All his infectious workup, including blood cultures, were negative. Bone marrow biopsy showed normocellular marrow with left-shifted trilineage hematopoiesis, with a predominance of early erythroid and myeloid precursors and no increase in blasts or significant morphologic dysplasia. Cytogenetics and myelodysplastic syndrome panel were normal. His ANC started to improve by day four of prednisone and filgrastim (Fig. 1). From his serial blood samples, he also had T-cell counts and cytokines checked (Fig. 2). He had complete recovery of his neutrophil count with ANC of 2400/ μL by day six of admission. Computerized Tomography imaging of the chest done during admission showed intrathoracic disease response compared to the CT performed 6 weeks earlier. His steroid taper schedule was 80 mg daily for week 1, 40 mg daily for week 2, 20 mg daily for week 3, 10 mg daily for week 4 and then stopping. His pembrolizumab was kept on hold.

However, eight weeks after being discharged, he was readmitted again with fever, cough, and shortness of breath. He was again noted to be neutropenic with ANC of 0 / μL (Fig. 1). Other complete blood count parameters were within the normal range. His CRP had again increased to 43.5 mg/L, from 7.7 mg/L at the last clinic visit 6 weeks prior. He was started on broad-spectrum antibiotics, prednisone 1 mg/kg, and a single dose of peg-filgrastim. Viral studies (HIV, Hepatitis-B, Hepatitis-C, and CMV) were negative. EBV IgG and Parvovirus B19 IgG titers were elevated and thus consistent with prior infection. Antinuclear antibodies (ANA), antineutrophil cytoplasmic antibodies (ANCA), rheumatoid factor (RF), and neutrophil-associated antibodies were negative, and C3 and C4 levels were normal. His neutrophil counts recovered to > 1500 on day seven of the second hospitalization.

His neutrophil counts were 5440/ μL at his post-discharge clinic follow up a week later. A restaging PET scan showed continued response with some areas of complete metabolic response. He continued prednisone taper for 8 weeks. Although his ANC showed persistent recovery and remained > 5000 / μL after his second hospitalization, his pembrolizumab was kept on hold. Repeat PET scan 7 weeks after the second neutropenia showed the metabolic activity of a mass like consolidation with air bronchogram in the posterior right upper lung and moderate focal uptake in the enlarged portocaval lymph node and tiny periaortic lymph nodes suggesting recurrent malignancy with likely a post-obstructive pneumonia. Unfortunately, despite adequate outpatient management, he had recurrent episodes of bacterial pneumonia complicated by hospitalizations due to which his performance status declined considerably. Due to this, he could not be initiated on any further treatment. Three months after his second neutropenic episode, he died from hypoxemic respiratory failure secondary to bacterial pneumonia that was unrelated to ICI use or neutropenia.

## Discussion

Neutropenia, as an irAE secondary to ICI is a rare finding with no comprehensive reports or clear management guidelines published to date. Here we discuss a unique case of isolated neutropenia secondary to ICI and also summarize previously reported cases with similar findings published in the literature. The absence of confounders, such as recent chemotherapy or medications that can cause cytopenias strongly supports our diagnosis of ICI as the primary etiology for the neutropenia. Furthermore, from a biomarker standpoint, we have also attempted to correlate serial inflammatory markers derived from blood and plasma with the ICI treatment course and the occurrence of neutropenia, an aspect that has not been documented to date.

Although immunotherapy has revolutionized the management of several tumor types, the occurrence of irAEs as a side effect can lead to significant morbidity as well as premature treatment discontinuations. Currently, the putative relationship between anti-tumor immunity and irAEs is not well understood. IrAEs are believed to be related to ICI mediated alterations in the roles that immune checkpoints play in maintaining immunologic homeostasis leading to the generation of auto-inflammatory responses [18]. Thus irAEs are more likely to reflect an exaggerated host immune function. Both auto-reactive T-cell and antibody-mediated processes have been speculated to mediate irAEs [6]. These theories are supported by some emerging data demonstrating T-cell clone cross-reactivity with antigens/epitopes shared between tumors and healthy tissue in patients presenting with irAEs [19, 20]. Also, alterations in various B-cell subsets correlating with timing and incidence of irAEs has been observed [21]. Similar to other irAEs, the mechanisms proposed for hematologic toxicities include generation of autoreactive T and B-cells as well as a decrease in T-regulatory phenotype [7].

The first neutropenia occurrence in our patient was after 4 cycles of pembrolizumab. This is similar to other reported cases (Table 1) where the median time to onset of neutropenia was after 3 cycles (range 2–11). Based on our literature review, the median time to onset of hematological toxicities has been noted to be shorter for anti-CTLA-4 monotherapy or the combination anti-CTLA-4/anti-PD-1 therapy compared to anti-PD-1 therapy alone [8]. In a majority of the cases we have reported (Table 1), and in nine other patients in the French study [9], nivolumab was the most common ICI resulting in neutropenia. However, given the limited number of patients, establishing a causal relationship between a specific ICI and neutropenia is not possible. A significant majority of documented cases (Table 1), including data reported from the French registry, were grade-4. Per the French study, more than 60% of isolated neutropenias were associated with febrility, which corresponds to our patient’s presentation [9]. Most of the patients in the previously published cases had other concurrent irAEs that manifested as rash, hepatitis, and colitis (Table 1). Based on data from 168 hematological toxicities observed in the WHO VigiBase, around 23% had concurrent non-hematological toxicities [8]. Our patient, however, presented with isolated neutropenia and no other accompanying irAEs. Due to the scarcity of data on ICI related neutropenia, for now, it is unclear which concurrent non-hematological irAEs have a stronger association with neutropenia and whether outcomes differ among these irAE subsets.

Due to the severity of the index neutropenic event, neither our patient nor any of the other reported patients were resumed on ICI. This permanent discontinuation of ICI broadly conforms to recent treatment guidelines for irAEs published by the American Society of Clinical Oncology where all leukopenias have been grouped as a single entity [22]. Intriguingly, our patient’s course was complicated by a recurrence of severe neutropenia despite holding ICI and complete resolution of the first neutropenic episode. Although evidence from literature supports that a majority irAEs occur within the first 5–15 weeks of starting ICI, similar to our case there are some reports of late-onset toxicities both in the setting of ongoing immunotherapy and after stopping treatment [23, 24]. Durable responses have been linked to ICI induced persistent CD8^+^ T effector memory subset against tumor cells [25]. The potential cross-reactivity of these T-cells against normal tissue after stopping treatment is one of the plausible mechanisms that has been suggested to contribute to this phenomenon [18]. In addition, following infusion anti-PD-1 antibodies have a prolonged receptor occupancy of > 2 months on T-cells and a half-life that spans three to four weeks with a steady state concentration achieved in 19 weeks [26, 27]. We speculate all these factors in conjunction contributed to the delayed recurrence of neutropenia in our patient. A two-month post neutropenia PET in our patient showed ongoing near complete metabolic response despite treatment discontinuation. This ongoing response despite stopping pembrolizumab after neutropenia conforms to the premise of emerging data suggesting that irAEs may act as a marker of ongoing anti-tumor activity and benefit from ICI [28]. However, the patient passed away due to unrelated hypoxemia secondary to pneumonia.

Bone marrow evaluation of our patient did not reveal involvement with the underlying malignancy but demonstrated a normocellular marrow with left-shifted trilineage hematopoiesis. A majority of previously reported cases also underwent bone marrow biopsy demonstrating variable findings (Table 1). Our findings of a normocellular BM in the setting of severe neutropenia raises suspicion of ICI induced peripheral destruction of neutrophils. However, given the limited scope of our serological and biomarker studies, we could not establish if this were a T-cell or an antibody driven process. Nevertheless, in the context of ICI induced cytopenias that persist despite treatment discontinuation and immunosuppressive strategies, it is essential to rule out bone marrow etiologies, including obtaining genetic panels for myeloid disorders, especially in the elderly.

**Fig. 3:**
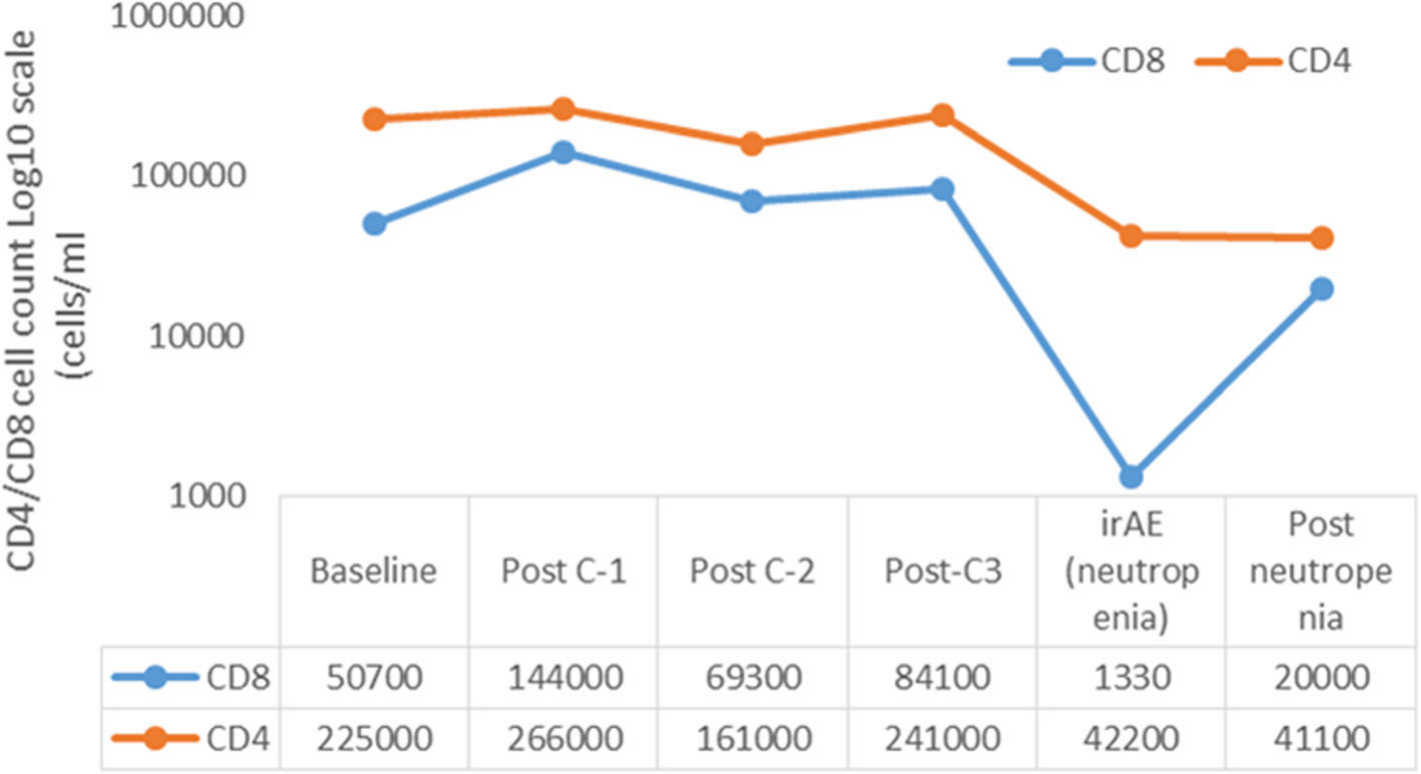
Changes in CD-4 and CD-8 cell counts during immunotherapy. Serial CD4/CD8 T –cell counts were obtained from peripheral blood and are plotted on a Log^10^ scale showing changes during immunotherapy course. Post-C2 refers to the sample collected on the day of C3 day-1 before anti-PD-1 administration. Post C3 refers to the sample collected on the day of C4 day-1 before anti-PD-1 administration and also represents the sample obtained prior to hospitalization due to neutropenia. Post-C3, when compared to post-C2, was noted to have a 1.2 and 1.5 fold increase for CD8 and CD4 counts, respectively. Drop in CD4/CD8 cell counts at neutropenia was likely because the in-hospital sample collection was after treatment with immunosuppression. Post neutropenia sample collection was at clinic follow up after discharge

Elevation in C-reactive protein (CRP) with neutropenia and subsequent fall with neutropenia resolution was a unique observation in our patient. We have previously reported on findings of elevations in CRP during irAEs compared to baseline levels before starting ICI [29]. Furthermore, we have also shown elevated levels of interleukin-6 (IL-6) corresponding to elevated CRP in a patient with pneumonitis [30]. This observation was again reproduced in the current patient where neutropenia corresponded to an elevation of not only IL-6 but also elevations in IL-17 and IL-10 levels (Fig. 2) compared to baseline. Altered levels of baseline cytokines/chemokines, including IL-6/IL-10, have also been described by others where lower baseline levels followed by a subsequent increase during treatment was seen in patients with irAEs [31]. Furthermore we have recently reported on the use of tocilizumab, an anti-IL-6 receptor antibody in the context of steroid-refractory irAEs, where we observed a significant benefit in terms of resolution of irAE symptoms and duration of hospitalization [32]. Although these observations are indirectly suggestive of altered cytokine physiology in promoting an immune dysregulation during irAEs, prospective validation to account for confounding etiologies (potential infection in our patient) that can contribute to dysregulation of cytokines is required. It is also important to note that both CD4 and CD8 cell counts were higher on the post-cycle-3 blood draw compared to post-cycle-2 (Fig. 3). A sustained and early rise in tumor-specific CD8 has been seen to co-relate with benefit from ICI [33]. This makes an argument that in addition to denoting ICI benefit, a rise in specific T-cell subsets beyond a critical threshold compared to baseline or significant alterations between subsequent cycles could be a marker for impending immune dysregulation leading to irAE. Thus understanding the relationships between T-cell subsets, cytokines, and irAEs in larger cohorts could be critical in identifying biomarkers for early detection of irAEs and choosing optimal candidates for ICI.

Due to its rarity, the optimal management strategy for ICI-related neutropenia is not established. As with most irAEs, response to immunosuppression after stopping the ICI has been documented in most of the published case reports (Table 1). Various combinations of high-dose steroidal and non-steroidal immunosuppression (cyclosporine, anti-thymocyte globulin, and mycophenolate mofetil) in addition to G-CSF or IVIG have been used to manage ICI-related neutropenia (Table 1). Despite some concern due to the potential of exacerbating underlying bacterial or fungal infections, steroid use has been consistently reported as part of initial management of ICI induced neutropenia. We recommend a slow tape of steroids after the index event to decrease chances of cyclic/recurrent exacerbation of neutropenia even after ICI has been discontinued. Thus, initiating prompt treatment can help in reducing the duration of neutropenia and thus prevent potentially life-threatening consequences.

## Conclusion

With rapid advances in the field of immuno-oncology and frequent use of newer ICI for multiple indications, we speculate that the potential for encountering unique irAEs secondary to ICI will rise. Our case adds to the growing body of evidence alluding to the unique immune adverse effect profiles of ICI. Evidence from our review establishes that ICI related neutropenia, although rare, tends to be severe, with a majority being grade-4. These immune-mediated neutropenias can lead to significant morbidity and mortality arising from infectious complications. Permanent ICI discontinuation needs to be strongly considered in almost all patients. Hence ICI related neutropenia as an irAE requires early identification with prompt interventions using immune suppression and granulocyte colony-stimulating factors to perhaps mitigate duration and thus prevent potentially fatal outcomes.

## Data Availability

Data sharing does not apply to this article as no datasets were generated or analyzed during the current study.
